# Extended-spectrum beta-lactamase-producing strains among diarrhoeagenic *Escherichia coli*—prospective traveller study with literature review

**DOI:** 10.1093/jtm/taab042

**Published:** 2021-04-08

**Authors:** Anu Kantele, Tinja Lääveri

**Keywords:** Antimicrobial resistance, multidrug resistance, rifamixin, azithromycin, fluoroquinolone, travelers`diarrhea, ESBL

## Abstract

**Background:**

Antibiotics are no longer the primary approach for treating all travellers’ diarrhoea (TD): most cases resolve without antibiotics and using them predisposes to colonization by multidrug-resistant bacteria. Data are accumulating on increasing resistance among TD pathogens, yet research into the most common agents, diarrhoeagenic *Escherichia coli* (DEC), remains limited.

**Methods:**

A total of 413 travellers to the (sub)tropics were analyzed for travel-acquired diarrhoeal pathogens and ESBL-PE. To identify ESBL-producing DEC, ESBL-producing *E. coli* (ESBL-EC) isolates were subjected to multiplex qPCR for various DEC pathotypes: enteroaggregative (EAEC), enteropathogenic (EPEC), enterotoxigenic (ETEC), enteroinvasive (EIEC) and enterohaemorrhagic (EHEC) *E. coli*.

For a literature review, we screened studies among travellers and locals in low- and middle-income countries (LMICs) on the frequency of ESBL-producing DEC, and among travellers, also DEC with resistance to ciprofloxacin, azithromycin, and rifamycin derivatives.

**Results:**

Our rate of ESBL-EC among all DEC findings was 2.7% (13/475); among EAEC 5.7% (10/175), EPEC 1.1% (2/180), ETEC 1.3% (1/80) and EHEC (0/35) or EIEC 0% (0/5). The literature search yielded three studies reporting ESBL-EC frequency and thirteen exploring resistance to TD antibiotics among travel-acquired DEC. For EAEC and ETEC, the ESBL-EC rates were 10–13% and 14–15%, resistance to fluoroquinolones 0–42% and 0–40%, azithromycin 0–29% and 0–61%, and rifaximin 0% and 0–20%. The highest rates were from the most recent collections. Proportions of ESBL-producing DEC also appear to be increasing among locals in LMICs and even carbapenemase-producing DEC were reported.

**Conclusion:**

ESBL producers are no longer rare among DEC, and the overall resistance to various antibiotics is increasing. The data predict decreasing efficacy of antibiotic treatment, threatening its benefits, for disadvantages still prevail when efficacy is lost.

## Introduction

Uncontrolled use of antibiotics is a major driver of the ongoing antimicrobial resistance (AMR) pandemic, which threatens global health.^1^ Increasing fastest in the tropics,^1^ AMR is being transported worldwide by international travellers: 20–70% of visitors to low- and middle-income countries (LMICs) carry multidrug-resistant bacteria (MDR), particularly extended-spectrum beta-lactamase-producing *Enterobacteriaceae* (ESBL-PE), to their home country^2–7^ and may spread them further.^2^^,^^6^ During the past decade, avoiding unnecessary antibiotic use while abroad has emerged as a means to combat travel-related global spread of AMR. In addition to the general pressure to avoid unnecessary antibiotics,^1^^,^^8^ this policy is particularly encouraged by findings that antibiotic use predisposes travellers to acquisition of multidrug-resistant intestinal bacteria^2–7^—and thus contributes to the global spread of AMR, colonized travellers acting as intercontinental transporters.^7^^,^^9^^,^^10^

Special attention has been paid to treatment of travellers’ diarrhoea (TD), which ranks as the most common indication for travellers’ antibiotic use^11^: 5–45% of those with TD take these drugs to alleviate their symptoms.^3–6^^,^^12–17^ As described in the literature, stand-by antibiotics for TD are prescribed at pre-travel consultations for 7–20% of European^11^^,^^13^^,^^15^ and practically all US travellers.^12^^,^^16^^,^^17^ Recently, the rates have also decreased somewhat in the USA.^18^ While antibiotics certainly retain their place in treating the most severe TD cases, their use for moderate TD has recently become topical.^19^ Although compared to placebo, antibiotics shorten the disease duration by 0.7–1.5 days,^20^^,^^21^ in most TD cases, the drugs are not necessary, since the disease usually resolves spontaneously. Anti-diarrhoeals such as loperamide offer an alternative with no impact on AMR colonization^22^; there are no studies that prove antibiotics to be clinically superior to loperamide in treatment of mild/moderate TD.^22^

In discussions concerning antibiotics for TD,^11^^,^^23–25^ limited attention has been given to resistance among diarrhoeagenic *Escherichia coli* (DEC), the most common TD pathogens^26^; studies have mainly examined *Salmonella*, *Campylobacter* and *Shigella.*^27–33^ DEC include several pathotypes: enteroaggregative (EAEC), enteropathogenic (EPEC), enterotoxigenic (ETEC), enteroinvasive (EIEC), enterohaemorrhagic (EHEC) or shiga-toxin-producing (STEC) *E. coli.*^34^ The paucity of resistance studies can be explained by the challenges in detecting the various DEC: as they in culture resemble any other *E. coli*, identifying a specific DEC type requires additional screening by PCR or other methods.^35^

Resistance has been reported among DEC in LMICs against the antibiotics currently recommended for TD treatment, but for travel-acquired DEC, the rates are only provided by a few studies. Ouyang-Latimer et al. showed already 2011 a substantial increase in MIC values for ciprofloxacin and azithromycin between 1997 and 2006–08 among both EAEC and ETEC isolates from travellers to Mexico, Guatemala and India.^36^ Moreover, travel-acquired ESBL-EAEC and ESBL–ETEC have been detected.^37–39^ ESBL-DEC are of special interest, since for severely ill travellers hospitalized, first-line intravenous drugs include third-generation cephalosporins (3GC) ineffective against these pathogens.^40^ Emergence of MDR strains among DEC is not unexpected—a similar development has been reported for other stool bacteria such as *Salmonellae.*^28^ Scarcity of research into travel-acquired ESBL-DEC prompted us to revisit our data on 413 Finnish travellers to investigate the frequency of ESBL producers among various DEC. Since our samples were collected ten years ago and the global AMR situation is constantly deteriorating, to get a more accurate picture, we also screened the literature for investigations into ESBL producers, resistance of travel-acquired DEC to commonly used antibiotics, and rates of ESBL-DEC among locals in LMICs. Research into the resistance of TD pathogens provides fundamental information for guidance on antibiotic treatment of TD.

## Materials and Methods

**Figure 1:**
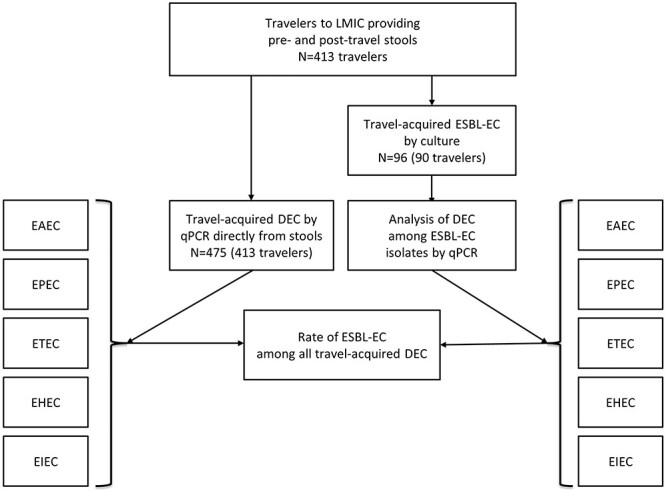
Flow chart of prospective study of ESBL-EC (extended-spectrum beta-lactamase-producing *Escherichia coli*) rates among DEC (diarrhoeagenic *E. coli*) of various pathotypes. Abbreviations: EAEC—enteroaggregative *E. coli*, EPEC—enteropathogenic *E. coli*, ETEC—enterotoxigenic *E. coli*, EIEC—enteroinvasive *E. coli*, EHEC enterohaemorrhagic *E. coli* or STEC—shiga-toxin-producing (STEC) *E. coli*.

The first part of this two-faceted study explored the rates and geographic origin of ESBL-producing strains among DEC contracted by Finnish travellers to LMICs (Figure 1). The second part searched PubMed for original studies of DEC exploring proportions of ESBL producers (travellers and locals) and resistance to commonly used TD antibiotics (only travellers).

### Study design, volunteers, samples and travel destinations

We prospectively recruited 526 Finnish travellers attending pre-travel consultation at the Travel Clinic of Aava Medical Centre before their journey outside the Nordic countries for more than four nights.^3^ Of these, 413 met our inclusion criteria (provided pre- and post-travel stools, filled in pre- and post-travel questionnaires, travel destination in LMICs). The details of stool collection, questionnaires and categorization of travel destinations have been described in our previous study.^3^

Post-travel ESBL-producing *Enterobacteriaceae* (ESBL-PE) were considered as travel acquired only if pre-travel samples had been negative for ESBL-PE.

The protocol was approved by the Helsinki University Hospital ethics committee. All subjects provided written informed consent.

### Collection of specimens

Briefly, faecal samples were collected before departure and from the first or second stools passed after returning home. For collection, we used swabs in Copan M40 Transystem tubes (Copan Diagnostics, Brescia, Italy). Once the samples arrived, total nucleic acids were extracted using the standard semiautomated protocol of easyMAG (bioMérieux, Marcy l’Etoile, France) and the stools were cultured (see below).

### Identification of ESBL-PE

As described earlier,^3^ ESBL-PE were isolated and characterized using established methods with culture on chromID ESBL (BioMérieux, Marcy-l’étoile, France), followed by double-disk synergy (Oxoid, Thermo Fisher Scientific, Hampshire, UK) test for cefotaxime, ceftazidime and cefpodoxime (30 μg each), alone or with clavulanic acid (10 μg), and species identification by Vitek GN (BioMérieux). Susceptibility testing for ciprofloxacin, cotrimoxazole, nitrofurantoin, tobramycin, ertapenem, imipenem and meropenem was conducted with E-test (BioMérieux) according to criteria set by the European Committee on Antimicrobial Susceptibility Testing EUCAST 5.0 (2018; www.eucast.org). Finally, beta-lactamase genes (TEM, OXA, SHV, CTX-M) and plasmid-mediated AmpC beta-lactamase genes (DHA, CIT) were identified by multiplex PCR.^41^ The co-resistance rates,^42^ prevalence of beta-lactamase genes,^3^ and phylogroup characterization^43^ of the ESBL-PE strains have been reported in our previous papers.

### Analysis of DEC by qPCR

To explore the proportion of ESBL producers among various DEC (Figure 1), we first explored the total rates of stool samples positive for DEC by a multiplex qPCR assay, which identifies nine bacterial pathogens: *Salmonella, Yersinia, Campylobacter, Vibrio cholerae, Shigella*/EIEC, EHEC, ETEC, EAEC and EPEC.^44^ Second, to identify ESBL-DEC in the same samples, we subjected the ESBL-EC isolates to the multiplex qPCR for DEC.

### Search for articles in PubMed

We searched PubMed for ‘ESBL’ or ‘extended-spectrum beta-lactamase’ or ‘CTX’ combined with ‘diarrh(o)eagenic’, ‘enteroaggregative’, ‘enteropathogenic’, ‘enterotoxigenic’, ‘enteroinvasive’, ‘enteroh(a)emorrhagic’, ‘shiga-toxin-producing’ or ‘verocytoxigenic’, ‘DEC’, ‘ETEC’, ‘EAEC’, ‘EPEC’, ‘EIEC’,‘EHEC’, ‘STEC’ or ‘VTEC’ and ‘est’, ‘elt’, ‘eae’, ‘aggR’, ‘bfpA’, ‘ipaH’ and ‘stx’, plus selected articles in our own collections that reported ESBL-production among the various DEC in human samples. Although *Shigella* and EIEC often cannot be distinguished by qPCR, we did not collect resistance data from studies reporting the ESBL-producing strains as *Shigella*.

## Results

### Participants

Demographics of the 13 with travel-acquired ESBL-DEC are provided in Table 1. Of them, 12/13 (92%) had TD and 2/12 (17%) took antibiotics for it. The entire study cohort’s demographics have been published earlier^3^; 67% had TD, 12% took antibiotics for it and 21% (90/430) were colonized by travel-acquired ESBL-PE (none of the travellers had ESBL-DEC in their pre-travel stools).

**Table 1:** Demographics of 13 prospectively recruited travellers who contracted extended-spectrum beta-lactamase-producing diarrhoeagenic *Escherichia coli* (ESBL-DEC) during visits to low- and middle-income countries (LMICs)

Age (years)	Gender	Type of ESBL-DEC	Concomitant other ESBL-PE	AB use	TD	Travel destination(s)	Length of travel (days)	Non-ESBL co-pathogens
23	Male	EAEC		No	Yes	Laos, Cambodia, Vietnam	22	None
31	Female	EPEC		No	Yes	India	11	EAEC, *Campylobacter*
61	Female	EPEC		FQ	Yes	China	12	ETEC
56	Female	EAEC		No	Yes	India	7	EPEC ETEC
67	Male	EAEC		No	No	Egypt, Jordan	7	None
24	Female	EAEC		No	Yes	Thailand, Cambodia, Vietnam	110	EPEC, *Campylobacter*
46	Female	EAEC	Non-DEC *E. coli*	No	Yes	Cambodia	19	EPEC
47	Male	EAEC		No	Yes	India	16	EHEC
22	Female	ETEC		No	Yes	India	14	EPEC EAEC
20	Male	EAEC	*Klebsiella pneumoniae*	FQ	Yes	India	16	EPEC, *Salmonella,* *Campylobacter*
31	Male	EAEC		No	Yes	India	27	EPEC *Campylobacter*
25	Male	EAEC	*E. hermannii*	No	Yes	India	32	EPEC
59	Male	EAEC		No	Yes	India	13	EPEC

Data are provided for concomitant other ESBL-producing *Enterobacteriaceae* (ESBL-PE), antibiotic (AB) use, travellers’ diarrhoea (TD), destination, length of travel and non-ESBL-PE co-pathogens.

Eight of the 13 participants with ESBL-DEC (61.5%) had travelled to South Asia, and three (23.1%) to the Southeast Asia. None of the visitors to sub-Saharan Africa or Latin America had ESBL-DEC.

### ESBL producers among DEC

The rate of ESBL-EC was 2.7% (13/475) among all DEC strains; 5.7% (10/175) among EAEC, 1.1% (2/180) among EPEC, 1.3% (1/80) among ETEC and 0% among EHEC (0/35) or *Shigella*/EIEC (0/5) strains (Table 2). EIEC and *Shigella* are indistinguishable in the qPCR assay, but as the same samples proved negative in *Shigella* culture, the isolates were considered as EIEC.

**Table 2:** Proportions of ESBL-producing *Escherichia coli* (ESBL-EC) among all DEC in samples from 413 travellers visiting LMICs

	n/all 90 ESBL-EC	ESBL-DEC /all respective DEC^a^	TD^b^	South Asia	South East Asia	East Asia	North Africa and Middle East	Sub-Saharan Africa	Latin America
				ESBL-DEC^c^	ESBL-DEC^c^	ESBL-DEC^c^	ESBL-DEC^c^	ESBL-DEC^c^	ESBL-DEC^c^
	*n* (%)	*n* (%)	*n* (%)	*n* (%)	*n* (%)	*n* (%)	*n* (%)	*n* (%)	*n* (%)
EAEC	10 (11.1)	10/175 (5.7)	9 (90.0)	6/33 (18.2)	3/33 (9.1)	0/1 (0.0)	1/3 (33.3)	0/90 (0.0)	0/15 (0.0)
EPEC	2 (2.2)	2/180 (1.1)	2 (100.0)	1/30 (3.3)	0/44 (0.0)	1/2 (50.0)	0/4 (0.0)	0/83 (0.0)	0/17 (0.0)
ETEC	1 (1.1)	1/80 (1.3)	1 (100.0)	1/12 (8.3)	0/19 (0.0)	0/0 (0.0)	0/0 (0.0)	0/45 (0.0)	0/4 (0.0)
Total	13 (14.4)	13/475 (2.7)	12 (92.3)	8 (62.0)	3 (23.1)	1 (7.7)	1 (7.7)	0 (0.0)	0 (0.0)

^a^ESBL producers (*n*) among all EAEC/EPEC/ETEC/DEC of 413 travellers (%)

^b^among 13 travellers with ESBL-DEC

^c^ESBL producers (*n*) among all EAEC/EPEC/ETEC/DEC in samples of travellers to region (%)

DEC were determined by multiplex qPCR directly from stools; positive result was interpreted as one strain. ESBL-DEC were identified by qPCR analysis of isolates initially obtained by culture. Table shows prevalences of various ESBL-DEC among all DEC strains (total = 475) of same type plus geographic origin as judged from stools of travellers visiting each region

Among strains originating in South Asia, 8.3% (1/12) of ETEC and 3.3% (1/30) of EPEC produced ESBL. The highest frequencies of ESBL-EAEC were seen for South Asia (6/33; 18.2%), the Southeast Asia (3/33; 9.1%) and North Africa and the Middle East (1/3; 33.3%).

Two volunteers had taken antibiotics (ciprofloxacin) for TD; both had an ESBL-DEC co-resistant to ciprofloxacin and tobramycin, whereas among those without antibiotic use, only one strain (1/11; 9.1%) was co-resistant to ciprofloxacin (Supplementary Table 1).

### ESBL genes

A total of 8/13 (61.5%) of the ESBL-DEC had *bla*_CTX-M-15_. The genes characterized for the nine ESBL-EAEC strains were *bla*_CTX-M-1_ (5/9), *bla*_CTX-M-9_ (3/9), *bla*_TEM_ (4/9), and *bla*_SHV_ (1/9); for the two ESBL-EPEC strains *bla*_TEM_ (2/2) and *bla*_CTX-M-1_ (2/2); and the only ESBL-ETEC *bla*_CTX-M-1_ (1/1) (Supplementary Table 2). Six of nine ESBL-DEC harboured genes of two types.

### Literature on resistance among DEC, special focus on rates of ESBL-DEC

In our literature search for studies of ESBL-DEC, we omitted those not reporting total number of DEC^43^^,^^45^ or strain-specific travel data^46^^,^^47^; these reports prove existence of ESBL-DEC, though. Instead, we selected, in accord with our initial aim, papers providing prevalence data on resistance among travel-acquired DEC or rates of ESBL-DEC among DEC originating in LMICs. Due to meagre search results especially among travellers, we also reviewed our own files on TD studies.

Our search only yielded 24 original studies of ESBL-DEC rates among one or more types of DEC, three traveller studies^37–39^ (Table 3), and 21 looking at locals in LMICs^48–68^ (Table 4). As for travellers, we found four other investigations into resistance rates to 3GC.^36^^,^^69–71^ In total, 13 traveller studies provided resistance rates to one or more TD antibiotics,^30^^,^^36–39^^,^^69–76^ all presented below by DEC pathotype.

**Table 3:** Results of literature search for traveller studies exploring antibiotic resistance among various DEC

First author year	Year(s) of stool sampling	Population, number of isolates	ESBL-EC	Cipro-floxacin resistance	Azithro-mycin resistance	Rifaximin resistance
Lurchachaiwong 2020^71^	2013–17	US military, Thailand ETEC 3 EAEC 3 EPEC 13 EIEC 1	Only resistance to ceftriaxone tested 0%	ETEC 0% EAEC 0% EPEC 8% EIEC 0%	NT	NT
Murphy 2019^76^	2012–14	Travellers in Nepal ETEC 60 EAEC 208 EPEC 65 EIEC 10	NT	ETEC 23% EAEC 15% EPEC 23% EIEC 10%	ETEC 22% EAEC 61% EPEC 67% EIEC 30%	NT
Guiral 2019^39^	2011–17	TD Spain ETEC 43 EAEC 39	ETEC 14% EAEC 13%	ETEC 33% EAEC 42%	ETEC 29% EAEC 33%	ETEC 0% EAEC 0%
Margulieux 2018^38^	2001–16	Locals and travellers, Kathmandu, Nepal ETEC 265	ETEC 15%	ETEC 6%	NT	NT
Mason 2017^30^	2002–04	US military, Thailand ETEC 29 EAEC 5 EPEC 16	NT	ETEC: 0% EAEC 0% EPEC 0%	ETEC: 0% EAEC 40% EPEC 13%	NT
Jennings 2017^71^	2003–10	Language school travellers, Cuzco, Peru ETEC 27 EAEC 9	ETEC 0% EAEC 11% nonsusceptible to ceftriaxone	ETEC: 0% EAEC: 7%	ETEC: 22% EAEC 33%	NT
Pandey 2011^75^	2001–03	Travellers and expatriates, Nepal ETEC 50 EPEC 38	NT	ETEC 0% EPEC10%	ETEC 16% EPEC 37%	NT
Guiral 2011^37^	2005–06	Spanish travellers to India with TD EAEC 51	EAEC 10%	Not reported	Not reported	Not reported
Ouyang-Latimer 2011^36^	2006–08	TD among travellers to Mexico, Guatemala, India ETEC 365 EAEC 26 India ETEC 98 EAEC 3 Mexico, Guatemala ETEC 270 EAEC 20	Resistance to ceftriaxone India ETEC 6% EAEC 0% Mexico, Guatemala ETEC 5% EAEC 20%	India ETEC 28% EAEC 0% Mexico, Guatemala ETEC 18% EAEC 35%	India ETEC 25% EAEC 0% Mexico, Guatemala ETEC 16% EAEC 40%	India ETEC 20% EAEC 0% Mexico, Guatemala ETEC 16% EAEC 0%
Porter 2010^74^	2002	US military, Turkey ETEC 82	NT	ETEC 5%	Not reported	NT
Mendez 2009^73^	1994–97 and 2001–04	Spanish travellers 1994–97 ETEC 82 EAEC 50 2001–04 ETEC 108 EAEC 54	NT	1994–97 ETEC 1% EAEC 2% 2001–04 ETEC 8% EAEC4%	NT	NT
Gomi 2001^69^	1997	Travellers to India, Mexico, Jamaica, Kenya ETEC 97 EAEC 75	a	India ETEC 3/61 (4.9%) EAEC 4/44 (9.1%)	a	a
Vila 2000^72^	1994–97	Spanish travellers ETEC 82	NT	ETEC 1%	NT	NT

^a^Resistance rates for ETEC and EAEC only provided together; cases with both reported as ‘highly sensitive’.

Some studies were conducted among both travellers and locals in LMICs. Table combines results from analyses of ESBL-DEC and resistance to TD antibiotics, fluoroquinolones, azithromycin, and rifaximin. Three studies only report resistance rates to third-generation cephalosporins but not ESBL-DEC (NT = not tested).

**Table 4:** Results of literature search for studies exploring rates of ESBL producers among various DEC isolated from stools of locals in various regions in LMICs

First author year	Year(s) of stool sampling	Population, number of isolates	ESBL-EC	Carbapenem resistance	Ciprofloxacin resistance	Azithromycin resistance	Rifaximin resistance
South Asia							
Moharana 2019^65^	2012–17	Indian children with diarrhoea DEC 77	4%	3%	74%	NT	NT
Mandal 2017^60^	not reported (“during two consecutive years”)	Indian children with diarrhoea DEC 191	All DEC 38% ETEC 18% EAEC 7% EPEC 11% EIEC 100% EHEC 0%	0%	DEC 50% resistant to levofloxacin	NT	NT
Khalil 2016^56^	2010–11	Pakistani children with diarrhoea EAEC 35	34%	NT	69%	NT	NT
Younas 2016^55^	2010–12	Pakistani children EPEC 46	59%	NT	39%	NT	NT
Malvi 2015 ^53^	2012–13	Indian children with/without diarrhoea EPEC 59	25%	30%	25%	14%	NT
Southeast Asia							
Our search yielded no studies conducted in the Southeast Asia							
East Asia							
Xu 2018^63^	2006–15	Chinese patients with diarrhoea aEPEC 151	25%	0%	5%	NT	NT
Zhou 2018^64^	2015–16	Chinese children with diarrhoea DEC 54	52%	6%	50%	NT	NT
Wang 2015^54^	2015	Chinese healthy elderly (>65 years) EAEC 96	56%	NT	NT	NT	NT
North Africa and Middle East							
Farajzadeh-Sheikh 2020^68^	2016–17	Iranian children EIEC 13 (5.1% of all DEC strains) ; other DEC not specified	EIEC 69%	EIEC 15%	0%	NT	NT
Eltai 2020^67^	2017–18	Quatarian children EAEC 20 EPEC 56	EAEC; 20% EPEC: 23%	EAEC; 10% EPEC: 7%	0%	NT	NT
Taghadosi 2019^66^	2014–15	Iranian children ETEC 13 EPEC 26	ETEC 54% EPEC 62%	0%	ETEC 46% EPEC 19%	NT	NT
Mahdavi 2018^62^	2015–16	Iranian children with diarrhoea ETEC 6 EAEC 35 EPEC 10 EIEC 6	ETEC 100% EAEC 74% EPEC 90% EIEC 83%	(Imipenem) ETEC 50% EAEC 14% EPEC 40% EIEC 0%	ETEC 17% EAEC 20% EPEC 40% EIEC 0%	NT	NT
Amin 2018^61^	2015–16	Iranian children with diarrhoea EAEC 32	28%	9% resistant to meropenem; 0% to imipenem	19%	78%	NT
Aminshahidi 2017^57^	2014–15	Iranian children DEC 48	DEC 67% ETEC 75% EAEC 85% EPEC 33% EIEC 50%	0%	DEC 31% ETEC 25% EAEC 27% EPEC 33% EIEC 50%	NT	NT
Karami 2017^58^	Not reported	Iranian children with/without diarrhoea EPEC 192	80%	0%	21%	NT	NT
Memariani 2015^52^	2011–13	Iranian children with diarrhoea EPEC 42	21%	NT	17%	NT	NT
Ghorbani-Dalini 2015^51^	2010	Iranian adults with diarrhoea DEC 54; DEC types not specified	13%	6% resistant to imipenem	8%	NT	NT
Khoshvaght 2014^50^	2011–12	Iranian children with diarrhoea EAEC 36	53%	4% resistant to imipenem	16%	NT	NT
Sonnevend 2006^48^	2003–04	children and adults with and without diarrhoea, United Arab Emirates EAEC 44	11%	NT	NT	NT	NT
Sub-Saharan Africa							
Konate 2017^59^	2013–15	children with diarrhoea, Burkina Faso DEC 31	68%	16% resistant to imipenem	0%	NT	NT
South and Central America and the Caribbean							
Amaya 2011^49^	2005–06	Nicaraguan children DEC 332	diarrhoea: ETEC 5/64 (8%) EAEC:23/134 (17%) EPEC: 3/34 (9%) EHEC: 0/8 (0%) EIEC 0/1 (0%) no diarrhoea: ETEC 1/9 (11%) EAEC: 13/69 (19%) EPEC:0/13 (0%) EHEC 0/0 (0%)	0%	1%	NT	NT

From the same papers, resistance rates are given also for carbapenems, fluoroquinolones, azithromycin and rifaximin, if tested (NT = not tested).

### Resistance among EAEC strains

Eight traveller studies describe resistance among EAEC strains (Table 3). Guiral et al. report for Spanish travellers with TD ESBL-EAEC rates of 10% (among 51 EAEC isolates in 2005–06) and 13% (39 EAEC in 2011–17).^37^^,^^39^

Among samples from language school students in Peru (2003–10), 11% of the EAEC isolates proved resistant to 3GC,^70^ for travellers to Mexico/Guatemala and India the figures were 20 and 0%, respectively (2006–08),^36^ and for the US military in Thailand 0% (2013–17)^71^.

Among travel-acquired EAEC, resistance rates of 0–42% have been reported to fluoroquinolones (eight articles^30^^,^^36^^,^^39^^,^^69–71^^,^^73^^,^^76^); 0–61% to azithromycin (six articles^30^^,^^36^^,^^39^^,^^69^^,^^70^^,^^76^) and 0% to rifaximin (three articles^36^^,^^39^^,^^69^).

The seven LMICs investigations show rates of 11–85% for ESBL-EAEC among EAEC (Table 4).^48^^,^^50^^,^^54^^,^^56^^,^^57^^,^^61^^,^^62^

### Resistance among ETEC strains

We found 12 resistance studies of travel-acquired ETEC^30^^,^^36^^,^^38^^,^^39^^,^^69–76^ (Table 3). For ESBL-ETEC, a rate of 14% was reported among 43 ETEC isolates from Spanish travellers in 2011–17^37^ and a rate of 15% among 265 ETEC isolates (from travellers and locals) from Kathmandu, Nepal in 2001–16.^38^ Among the most recently acquired strains, the resistance rates amounted to 34–35%.^38^

Of the three studies reporting resistance to 3GC, a rate of 0% was recorded for language school students in Peru 2003–10^70^ and US military in Thailand,^71^ and 5 and 6% for travellers to Mexico/Guatemala and India, respectively, in 2006–08.^36^

Resistance among ETEC to fluoroquinolones was explored in 12 traveller studies, showing rates of 0–33%^30^^,^^36^^,^^38^^,^^39^^,^^69–76^; seven studies explored resistance to azithromycin with rates of 0–29%^30^^,^^36^^,^^39^^,^^69^^,^^70^^,^^75^^,^^76^ and three to rifaximin with rates of 0–20%.^36^^,^^39^^,^^69^

The three investigations among locals in LMICs showed among ESBL-ETEC rates of 18% in India^70^ and 75 and 100% in Iran^57^^,^^62^ (Table 4).

### Resistance among EPEC strains

Our search yielded four traveller studies of EPEC strains (Table 3). In Nepal 2001–03^75^ and 2012–14,^76^ ESBL-EPEC were not covered, but resistance rates of 10 and 23% to fluoroquinolones, and 37 and 67% to azithromycin, were seen, respectively. Among US military in Thailand in 2002–04, resistance rates (ESBL-EPEC not covered) of 0 and 13% were recorded to fluoroquinolones and azithromycin,^30^ and in 2013–17 8% to ciprofloxacin.^71^

Among locals the six studies reported rates of 11–80% for ESBL-EPEC^52^^,^^53^^,^^55^^,^^57^^,^^58^^,^^60^^,^^63^ (Table 4).

### Resistance among EHEC/STEC strains

None of the traveller studies reviewed provided rates of antibiotic resistance for EHEC/STEC isolates.

Amaya et al. did not find any ESBL-EC among eight EHEC strains from Nicaraguan children with diarrhoea^49^ (Table 4).

### Resistance among EIEC strains

Our search yielded two traveller studies of resistance looking at EIEC isolates: among samples from US military in Thailand 2013–17 no resistance was detected^71^ but in Nepal 2012–14, 10% of the EIEC strains proved resistant to ciprofloxacin and 30% to azithromycin.^76^

In LMICs, studies among local children with diarrhoea have found the few EIEC strains to be mostly ESBL producers.^49^^,^^57^^,^^60^^,^^62^^,^^68^

## Discussion

Despite the vast discussion around antibiotic use for treating TD, paradoxically scant attention has been paid to resistance among the most common TD pathogens, DEC. The handful of reports published mostly do not focus on travellers. Apart from resistance to individual antibiotics, multidrug resistance is increasingly common among intestinal bacteria in clinical samples worldwide, ESBL-PE ranking as the most prevalent MDR type.^77–79^ Our data together with those from a literature search for studies among travellers and locals in LMICs destinations show an emergence of ESBL producers among DEC.

### Rates of ESBL producers among DEC

Our rate, 3–7% of ESBL producers among the various DEC strains collected 2009–10, appears consistent with the three other traveller studies of ESBL-DEC: among Spanish travellers, the rates of ESBL-EAEC were 10% in 2005–6,^37^ and 12.8% in 2011–17.^39^ Among residents and travellers with acute diarrhoea in Kathmandu an increase from 1.5 to 35% was observed between 2008 and 2016.^38^ These data suggest increasing rates of ESBL producers among DEC.

We found more investigations into the ESBL-production of DEC among locals in LMICs than among travellers, with rates of positive findings varying by pathotype, time and destination between 0 and 80%.^48–68^ It should be noted that none of the analyses focused on the main tourist destinations in Southeast Asia, Africa, or South and Central America, and the Caribbean. In 18 of the 21 studies, the data were from local children with or without diarrhoea,^48–50^^,^^52^^,^^53^^,^^55–62^^,^^64–68^ highlighting the clinical concern related to resistance. Likewise, among locals, the highest rates were recorded over the most recent years, according with the steady global increase in the rates of ESBL-producing strains among all *E. coli* in clinical samples.^77–79^

Our search did not focus on carbapenemase-producing DEC, but we found 16 studies from LMICs reporting resistance rates of 0–50% to carbapenems among DEC.^49–68^ Our samples showed no carbapenemase-producing genes.^3^

### ESBL producers among various DEC

In our data, the ESBL-EC rates appeared higher among EAEC than EPEC and ETEC (5.7% versus 1.1% versus 1.3%). This accords with other traveller studies reporting ESBL-EAEC rates of 10%^37^ and 12.8%^39^ among travellers yet amounting to 85% for locals in Iran^57^ and 56% in China.^54^ Likewise, substantial rates (53 and 57%) of ESBL-EAEC have been reported among clinical EAEC isolates in England; yet they do not report which of the strains were travel-acquired nor their countries origin.^46^^,^^47^

For ESBL-ETEC, our rate, 1.3% (Table 2), was much lower than that found among Spanish travellers (14%)^39^ or in Nepal (15%).^38^ The top rates (75%) for non-travellers have been recorded among Iranian children.^57^

As for EPEC, we only identified two ESBL-EPEC strains (1.1%). None of the traveller studies reviewed covered ESBL-EPEC, but among locals rates as high as 80% have been reported in Iran,^58^ and 59% in Pakistan.^55^

We detected no ESBL-EC among EIEC and EHEC, neither did we find in the literature any other traveller studies exploring ESBL-EC of these pathotypes; only few investigations among locals report ESBL-EC for EIEC or EHEC.^49^^,^^57^^,^^60^^,^^62^^,^^68^

We found no more than two studies looking at the rates from the other angle, describing the rates of a given pathotype among travel-acquired ESBL-DEC: rates of 14% in 2009–10^43^ and 57% in 2017–18^45^ have been shown for ESBL-DEC.

### Geographic distribution of ESBL-DEC

Most of our ESBL-DEC originated in South Asia, which also proved to have the highest rates of ESBL-DEC among DEC: 18.2% of EAEC strains were ESBL producers. Indeed, South Asia also has exceptionally high-resistance rates among gram-negative bacteria in clinical samples^80^^,^^81^ and top ESBL-PE colonization rates among visitors.^2–6^^,^^82^^,^^83^ Our data agree with previous data showing higher resistance rates among EAEC strains from South or Southeast Asia (33.3%; 4/12) than those from Africa (6.3%; 1/16) and Latin America (0%; 0/11).^39^

### Resistance to commonly used TD antibiotics

While our own results centre around ESBL-DEC, we also reviewed the literature for data on resistance among travel-acquired DEC to commonly used TD antibiotics (fluoroquinolones, azithromycin and rifaximin). Recent traveller studies^39^^,^^76^ present alarming data: for EAEC strains resistance rates of 15–42%, 33–61% and 0% to fluoroquinolones, azithromycin and rifaximin, and for ETEC 23–33%, 22–29% and 0%, respectively.

### Resistance genes among DEC

Our data include thirteen ESBL-DEC isolates, with *bla*_CTX-M-1_ as the most common finding in genetic analyses_,_ followed by *bla*_TEM_. Only a small proportion of our strains carried the *bla*_CTX-M-15_ gene despite the worldwide spread of *E. coli* clone of sequence type 131 (ST131) carrying the CTX-M-15 ESBL both in clinical and non-clinical settings.^84^ In contrast, a previous traveller study^36^ reports a total of 11 ESBL-DEC strains, all harbouring either of the two genes *bla*_CTX-M-15_ or *bla*_CTX-M-27_. Likewise, from the samples of residents and travellers in Nepal,^38^  *bla*_CTX-M-15_ was detected in 80% of the ESBL-ETEC strains.

### Clinical implications

While ESBL-EC are considered resistant to 3GC (e.g. ceftriaxone), the resistance profile as such does not cover the most commonly used TD regimens, i.e. fluoroquinolones, azithromycin and rifaximin. Unfortunately, however, ESBL-producing strains often harbour co-resistance to other antibiotics, especially fluoroquinolones.^85^^,^^86^ Of our ESBL-DEC strains, 3/13 (23.1%) were co-resistant to fluoroquinolones, yet higher co-resistance rates have been reported among travel-acquired ESBL-PE in general, particularly for the South Asia^2^^,^^5^^,^^42^^,^^45^^,^^82^ and related to fluoroquinolone intake abroad.^42^ Indeed, ESBL-producing strains are of special concern, since in cases severe enough to require hospitalization empiric treatment often relies on either 3GC or fluoroquinolones.^40^

Interpreting the efficacy of various antibiotics is somewhat complicated, for faecal antibiotic levels tend to exceed the minimum inhibitory concentration (MIC).^23^ Furthermore, presence of antibiotics in stools, while indicating an antibiotic pressure to other intestinal bacteria, may also drive transfer of resistance genes to other *Enterobacteriaceae,* some of which are potential pathogens.^85^^,^^87^

An ineffective drug does not offer benefits, and yet retains its disadvantages. Although the adverse effects rate appears to be low,^88^ recently, for example, the US Food and Drug Administration has warned about some serious adverse effects of fluoroquinolones (e.g. tendinitis and prolonged QT interval) and azithromycin (e.g. prolonged QT interval),^89^^,^^90^ the most popular TD antibiotics. Furthermore, data are lacking on the suggested smaller impact of one-day antibiotic treatment on acquisition of MDR bacteria abroad. The adverse effect profile would favour rifamycins such as rifaximin. However, the drug is non-absorbable and should not be used in cases with fever and invasive disease—i.e. it does not meet the most important indications for antibiotics. We only found a few studies exploring resistance rates to rifaximin among DEC; Ouyang-Latimer et al. ^36^ reported 16–25% resistance rates among ETEC already in 2011.

### Limitations of our data

Firstly, collected 2009–10, our strains do not fully represent the current situation. Unfortunately, though, the same applies to the other traveller studies found in our search, only three of which provide data from a later time period.^38^^,^^39^^,^^76^ The increase in resistance recorded among locals suggests growing pressure also for travellers. Our data may thus present a slight underestimation, calling for updated surveillance.

Secondly, qPCR of stools cannot distinguish whether the samples contain one DEC strain or several of similar type. Likewise, in culturing ESBL-EC strains, those which appear phenotypically different are picked, and therefore strains may be missed that are similar or of only a slightly different phenotype, but genetically unlike. Fortunately, these sources of error may at least partly overcome one another.

Thirdly, in the various studies reviewed there are methodological differences (assessment of the various DEC, pre-analytical handling of the specimens, etc.); therefore, the data may not be fully comparable.

## Conclusions

ESBL-producing DEC are no longer rare, particularly in Asia. Among travel-acquired DEC, their rates appear fairly low as yet, but in many regions, increase is already seen among DEC isolated from locals with acute diarrhoea, also portending increase among travel-acquired DEC, many strains even to be carried by travellers to their countries. While antibiotics certainly retain their place in the treatment of the most severe TD cases, data showing increasing resistance among stool pathogens further encourage cutting back on use of antibiotics for TD, and opting for non-antibiotic alternatives for mild and moderate cases. After all, an ineffective drug, while obviously useless, retains all its disadvantages.

## Authors’ contributions

Study concept and design by A.K. and T.L.; acquisition of data by A.K.; literature review by T.L.; statistical analysis by T.L.; drafting the manuscript by A.K. and T.L. and final approval of version published by A.K. and T.L.

## Supplementary Material

Supplementary_table_1_ESBL-DEC_281220_submitted_taab042

Supplementary_table_2_ESBL-DEC_281220_submitted_taab042
