# Supplementary material for: Extended-spectrum beta-lactamase-producing strains among diarrhoeagenic Escherichia coli—prospective traveller study with literature review
Source: J Travel Med. 2021 Apr 8;29(1):taab042. doi: 10.1093/jtm/taab042 (PMC8763120; doi:10.1093/jtm/taab042)
Supplement: Supplementary_table_1_ESBL-DEC_281220_submitted_taab042 [file supplementary_table_1_esbl-dec_281220_submitted_taab042.docx]

**Supplementary Table 1**. Antibiotic resistance among ESBL-DEC strains. Data given as MIC-values and interpretation in SIR system.

| **ESBL-DEC** | **Travel destination(s)** | **ceftazidime** | | **ceftriaxone** | | **cefepime** | | **erta-penem** | | **mero-penem** | | **tobra-mycin** | | **cipro-floxacin** | | **tige-cycline** | | **nitro-furantoin** | | **piperacillin/tazobactam** | | **trimethoprim-sulfamethoxazole** | | **colistin** | | |
| --- | --- | --- | --- | --- | --- | --- | --- | --- | --- | --- | --- | --- | --- | --- | --- | --- | --- | --- | --- | --- | --- | --- | --- | --- | --- | --- |
| EAEC | Laos, Cambodia, Vietnam | 0.75 | S | >32 | R | 3 | I | 0.012 | S | 0.032 | S | 1 | S | 0.012 | S | 0.25 | S | 12 | S | 1.5 | S | >32 | R | 0.094 | S |  |
| EPEC | India | 8 | R | >32 | R | 4 | I | 0.006 | S | 0.023 | S | 0.75 | S | 0.064 | S | 0.094 | S | 4 | S | 0.75 | S | >32 | R | 0.064 | S |  |
| EPEC | China | 8 | R | >32 | R | 8 | R | 0.023 | S | 0.023 | S | 4 | I | >32 | R | 0.125 | S | 16 | S | 1.5 | S | >32 | R | 0.094 | S |  |
| EAEC | India | 0.5 | S | >32 | R | 2 | I | 0.012 | S | 0.19 | S | 0.75 | S | 0.032 | S | 1 | S | 12 | S | 2 | S | 0.19 | S | 0.19 | S |  |
| EAEC | Egypt, Jordan | 1.5 | I | >32 | R | 8 | R | 0.032 | S | 0.25 | S | 1 | S | 0.016 | S | 0.19 | S | 24 | S | 2 | S | >32 | R | 0.125 | S |  |
| EAEC | Thailand, Cambodia, Vietnam | 16 | R | >32 | R | 12 | R | 0.032 | S | 0.25 | S | 0.75 | S | 0.047 | S | 0.25 | S | 8 | S | 1.5 | S | >32 | R | 0.125 | S |  |
| EAEC | Cambodia | 0.19 | S | >32 | R | 2 | I | 0.008 | S | 0.023 | S | 0.75 | S | 0.094 | S | 0.19 | S | 8 | S | 0.38 | S | >32 | R | 0.125 | S |  |
| EAEC | India | 12 | R | >32 | R | 8 | R | 0.032 | S | 0.032 | S | 0.75 | S | >32 | R | 0.25 | S | 16 | S | 1.5 | S | >32 | R | 0.125 | S |  |
| ETEC | India | 8 | R | >32 | R | 8 | R | 0.064 | S | 0.023 | S | 8 | R | >32 | R | 0.25 | S | 16 | S | 6 | S | 0.047 | S | 0.125 | S |  |
| EAEC | India | 24 | R | >32 | R | 16 | R | 0.047 | S | 0.047 | S | 0.75 | S | 0.19 | S | 0.125 | S | 8 | S | 1 | S | 0.032 | S | 0.125 | S |  |
| EAEC | India | 16 | R | 2 | I | 1 | S | 0.016 | S | 0.023 | S | 0.5 | S | 0.19 | S | 0.19 | S | 12 | S | 1 | S | 0.032 | S | 0.094 | S |  |
| EAEC | India | 8 | R | >32 | R | 8 | R | 0.016 | S | 0.032 | S | 0.75 | S | 0.19 | S | 0.19 | S | 16 | S | 1.5 | S | >32 | R | 0.125 | S |  |
