# Supplementary material for: Extended-spectrum beta-lactamase-producing strains among diarrhoeagenic Escherichia coli—prospective traveller study with literature review
Source: J Travel Med. 2021 Apr 8;29(1):taab042. doi: 10.1093/jtm/taab042 (PMC8763120; doi:10.1093/jtm/taab042)
Supplement: Supplementary_table_2_ESBL-DEC_281220_submitted_taab042 [file supplementary_table_2_esbl-dec_281220_submitted_taab042.docx]

**Supplementary Table 2**. Resistance genes of ESBL-DEC strains isolated from post-travel samples of travelers to LMIC.

| **ESBL-DEC** | **Travel destination(s)** | **TEM** | **OXA** | **SHV** | **CTX-M-1** | **CTX-M-2** | **CTX-M-9** | **CTX-M-8/25** | **CTX-M-15** |
| --- | --- | --- | --- | --- | --- | --- | --- | --- | --- |
| EAEC | Laos, Cambodia, Vietnam | 0 | 0 | 0 | 0 | 0 | 1 | 0 | 0 |
| EPEC | India | 1 | 0 | 0 | 1 | 0 | 0 | 0 | 1 |
| EPEC | China | 1 | 0 | 0 | 1 | 0 | 0 | 0 | 1 |
| EAEC | India | 0 | 0 | 0 | 1 | 0 | 0 | 0 | 1 |
| EAEC | Egypt, Jordan | 1 | 0 | 0 | 0 | 0 | 1 | 0 | 0 |
| EAEC | Thailand, Cambodia, Vietnam | 1 | 0 | 0 | 1 | 0 | 0 | 0 | 1 |
| EAEC | Cambodia | 0 | 0 | 0 | 0 | 0 | 1 | 0 | 0 |
| EAEC | India | 0 | 0 | 0 | 1 | 0 | 0 | 0 | 1 |
| ETEC | India | 0 | 0 | 0 | 1 | 0 | 0 | 0 | 1 |
| EAEC | India | 0 | 0 | 0 | 1 | 0 | 0 | 0 | 1 |
| EAEC | India | 1 | 0 | 1 | 0 | 0 | 0 | 0 | 0 |
| EAEC | India | 1 | 0 | 0 | 1 | 0 | 0 | 0 | 1 |
